# Transitioning to home and beyond following stroke: a prospective cohort study of outcomes and needs

**DOI:** 10.1186/s12913-024-10820-8

**Published:** 2024-04-10

**Authors:** Geraldine O’Callaghan, Martin Fahy, Sigrid O’Meara, Mairead Chawke, Eithne Waldron, Marie Corry, Sinead Gallagher, Catriona Coyne, Julie Lynch, Emma Kennedy, Thomas Walsh, Hilary Cronin, Niamh Hannon, Clare Fallon, David J Williams, Peter Langhorne, Rose Galvin, Frances Horgan

**Affiliations:** 1 iPASTAR Collaborative Doctoral Award Programme, School of Physiotherapy, RCSI University of Medicine and Health Sciences, 123 St. Stephen’s Green, Dublin 2, Ireland; 2 iPASTAR Collaborative Doctoral Award Programme, RCSI Division of Population Health Sciences, RCSI University of Medicine and Health Sciences, 123 St. Stephen’s Green, Dublin 2, Ireland; 3 Early Supported Discharge Team for Stroke, Galway University Hospital, Newcastle Rd, H91 YR71 Galway, Ireland; 4 Acute Stroke Team, Regional Hospital Mullingar, N91 NA43 Co. Westmeath, Ireland; 5 Acute Stroke Team, Beaumont Hospital, D09V2N0 Dublin 9, Ireland; 6 Consultant Geriatrician / Stroke Physician, Stroke and Geriatric Medicine, Galway University Hospital, Newcastle Rd, H91 YR71 Galway, Ireland; 7 Consultant Geriatrician, Regional Hospital Mullingar, N91 NA43 Co. Westmeath, Ireland; 8 Consultant Stroke Physician, Stroke and Geriatric Medicine, Galway University Hospital, Newcastle Rd, H91 YR71 Galway, Ireland; 9 Consultant Geriatrician, General Internal Medicine Physician & RCSI Undergraduate Dean, Regional Hospital Mullingar, N91 NA43 Co. Westmeath, Ireland; 10 Department of Geriatric and Stroke Medicine and iPASTAR Collaborative Doctoral Award Programme, RSCI University of Medicine and Health Sciences and Beaumont Hospital, Dublin 9, Ireland; 11 School of Cardiovascular and Metabolic Health (SCMH), University of Glasgow, G12 8QQ Glasgow, Scotland; 12 School of Allied Health, Faculty of Education and Health Sciences, Ageing Research Centre, Health Research Institute, University of Limerick, V94 T9PX Limerick, Ireland

**Keywords:** Post-stroke transition hospital-to-home, Outcomes assessment, Needs assessment, Rehabilitation intervention, Community reintegration

## Abstract

**Introduction:**

Understanding of the needs of people with stroke at hospital discharge and in the first six-months is limited. This study aim was to profile and document the needs of people with stroke at hospital discharge to home and thereafter.

**Methods:**

A prospective cohort study recruiting individuals with stroke, from three hospitals, who transitioned home, either directly, through rehabilitation, or with early supported discharge teams. Their outcomes (global-health, cognition, function, quality of life, needs) were described using validated questionnaires and a needs survey, at 7–10 days, and at 3-, and 6-months, post-discharge.

**Results:**

72 patients were available at hospital discharge; mean age 70 (SD 13); 61% female; median NIHSS score of 4 (IQR 0–20). 62 (86%), 54 (75%), and 45 (63%) individuals were available respectively at each data collection time-point. Perceived disability was considerable at hospital discharge (51% with mRS ≥ 3), and while it improved at 3-months, it increased thereafter (35% with mRS ≥ 3 at 6-months). Mean physical health and social functioning were “fair” at hospital discharge and ongoing; while HR-QOL, although improved over time, remained impaired at 6-months (0.69+/-0.28). At 6-months cognitive impairment was present in 40%. Unmet needs included involvement in transition planning and care decisions, with ongoing rehabilitation, information, and support needs. The median number of unmet needs at discharge to home was four (range:1–9), and three (range:1–7) at 6-months.

**Conclusion:**

Stroke community reintegration is challenging for people with stroke and their families, with high levels of unmet need. Profiling outcomes and unmet needs for people with stroke at hospital-to-home transition and onwards are crucial for shaping the development of effective support interventions to be delivered at this juncture.

**ISRCTN registration:**

02/08/2022; ISRCTN44633579.

**Supplementary Information:**

The online version contains supplementary material available at 10.1186/s12913-024-10820-8.

## Introduction

Stroke is the second leading cause of death in Western Europe, and the leading cause of severe long-term adult disability [1, 2]. Following acute and sub-acute stroke management individuals are discharged home either, directly, after a period of inpatient rehabilitation, or with early supported discharge (ESD). Typically a ‘care transition’, from the acute hospital, rehabilitation setting or to ESD, to community services, such as primary care, social care, mental health, and health and wellbeing services, is required. The American Geriatric Society defines transitional care as “a set of actions designed to ensure the co-ordination and continuity of health care as patients transfer between different locations or different levels of care” [3]. The transition to home for people after stroke is often challenging. After structured stroke services, including ESD conclude, stroke patients and their families are frequently disappointed and frustrated as the concept of organised stroke care disappears [4].

International evidence describes poor long-term functional, cognitive, and psychological outcomes [5–7] for a significant proportion of people living with stroke, with substantial unmet needs particularly in areas of cognition, emotion, fatigue, and finances, and requirements for ongoing rehabilitation, stroke-related information and support and signposting [4, 8, 9]. While ESD is recommended as best-practice for those with mild to moderate disability, is associated with positive clinical and process outcomes [10, 11] and has high associated user satisfaction [12], it is not appropriate or available for all [13], and there remains uncertainty around support interventions that are effective at transition to home [14]. As such, care transitions, specifically hospital-to-home and life after stroke, are a focus for many national, European and international guidelines and programmes [15–18].

Care transitions are known to be a high risk scenario for patients and their family, leading to a risk of adverse events, rehospitalisation and dissatisfaction with services [19]. The first step in improving the transition from hospital to home is to generate a comprehensive understanding of the transitional and long-term needs of people with stroke. There exists a gap in our knowledge about the unmet needs at discharge to home, and in the first 6-months after stroke.

### Aim

The aim of this study was to profile and document the needs of people with stroke at hospital discharge to home and thereafter.

This research is part of a PhD in Population Health, and under the iPASTAR (improving Pathways for Acute STroke And Rehabilitation) programme. **Patient and public involvement (PPI)**, including people with stroke, caregivers and stroke advocates, partnered with the iPASTAR programme to develop the research question. The research team continued this partnership with a smaller panel of PPI “stroke champions”, people with stroke, in developing the study methodology, and in analysing and reporting the findings in a meaningful way to people with stroke and their families, health professionals, and policymakers.

## Method

### Study design

A prospective cohort study was employed and the Strengthening and the Reporting of Observational Studies and Epidemiology (STROBE) guidelines [20] and checklist (supplemental material Table I) were used to improve reporting quality. The study was guided by the Institute of Medicines analytical framework for quality health care [21].

### Ethics

#### Ethics approvals

were obtained (ref: C.A. 2739; ref: 22/04; ref: RRECB0622GOC), and the study was registered: ISRCTN44633579.

### Setting

The study partnered with two acute urban based hospitals (Site 1 & 2) and a regional hospital (Site 3) to profile stroke patients discharged home directly, home after inpatient rehabilitation, and home via ESD.

### Recruitment

Consecutive adults with acute stroke from participating hospitals were recruited by gatekeepers, between March and December 2022. Eligible participants received oral and written information about the study, and written consent was obtained from those cognitively competent and willing to consent, and able to communicate or be supported in communication.

### Procedure

Within 7–10 days of discharge to home (T0), and at 3- (T1) and 6-months (T2) post-discharge, structured interviews using questionnaires and surveys determined self-reported outcomes and needs. Participant preference for data collection was facilitated (home visits, phone interviews, or video-conferencing), while adhering to relevant Covid-19 guidelines.

### Data Collection

#### Participant characteristics

Participant’s name, date of birth, and contact details were provided by a gatekeeper, while a self-reported questionnaire, administered by the primary researcher, gathered information on gender, first stroke (yes/no), co-habiting status, employment, and pre-stroke homecare.

#### Disease-related data

Disease-related data including date and type of stroke (ischemia/haemorrhage), stroke severity on admission, reperfusion (thrombolysis/thrombectomy (yes/no)), and level of communication (aphasia (yes/no)) were furnished by the gatekeeper. The National Institute of Health Stroke Severity Scale (NIHSS), a validated and reliable tool [22, 23], evaluated stroke severity on admission: level of consciousness, vision and gaze, facial palsy, extremity weakness, limb ataxia, sensory loss, language, dysarthria, and neglect. Participants scored between 0 and 42, with higher scores indicating a more severe stroke.

#### Outcomes

Outcomes and needs data were collected by the primary researcher.

The PROMIS-10 short form, was used to assess global health. It comprises eight item domains assessing areas of health and functioning such as overall physical health, mental health, social health, pain, fatigue, and overall perceived quality of life (QOL)(healthmeasures.net). The scoring system allows each of the individual domains to be examined separately and be collated into two summary scores: Global Physical Health (GPH) and Global Mental Health (GMH), which are used to calculate t-scores of the respective domains. The general population reference norm is 50, with a standard deviation of 10. Additionally, two single questions ask about achievement of social function and general health. Single domain measurement extends from 1 to 5, except for pain where the scores are recalculated from a 10 point pain scale into the 1 to 5 measurement, with lower values reflecting a poorer outcome. PROMIS 10 is recommended by the International Consortium for Health Outcomes Measurement (ICHOM) for use as part of a standard set of outcome measures in stroke [24]; and exhibits acceptable performance in validity and reliability testing [25].

The simplified modified Rankin Scale (smRS), considered a reliable and valid measure of function in stroke [26], assessed the degree of disability at each time-point. Scored on a six-point Likert scale disability was categorised as: 0) no symptoms; (1) no significant disability despite symptoms; (2) slight disability; (3) moderate disability; (4) moderate/severe disability; and (5) severe disability.

Cognitive impairment was screened using the Telephone Montreal Cognitive Assessment Scale (T-MoCA) [27]. The T-MoCA consists of six domains: short time/work memory, attention, abstraction, concentration, language, and orientation to time, and is a valid and reliable screening tool [27]. It allows for greater data gathering flexibility because it does not present the same access restrictions that video-conferencing does, as users can participate through telephone without having more specialised technological equipment or skill. The total score ranges from 0 to 22 and is converted back to MOCA scores (0–30), with lower scores indicating more severe cognitive impairment.

Health-related quality of life (HR-QOL) was determined using The EuroQol 5 Dimenion-5 L (EQ5D-5L), which consists of five dimensions (mobility, self-care, usual activities, pain and discomfort, anxiety & depression), each of which has five severity levels that are described by statements appropriate to that dimension [28]. An EQ5D index score can be calculated, based on the responses to the EQ5D-5L questionnaire, to represent an overall assessment of an individual’s health status. The EQ5D-5L has been found to be valid for use as a generic health outcome measure in stroke [29].

### Needs

Self-reported needs were assessed using the UK Stroke Survivor Needs Survey [4]. It asks 44 closed questions regarding stroke, health after stroke, everyday living, employment and leisure, family, friends, and use of support groups, personal and household finances, other needs, and facilitators of recovery. Additional comments around needs were facilitated by an open-ended question at the end of the survey [4]. At T1 and T2, the UK Stroke Survivor Needs Assessment was used in its entirety, with Q2 and Q3 modified at T0 to reflect what is more appropriate to ask people with stroke immediately on discharge from hospital. For administration of the needs survey, participants were given the option of completing it as a google docs form, postal questionnaire (prepaid reply envelope), via video-conferencing, or in a face-to-face interview. Non-responders (google docs form, postal questionnaire) were contacted after approximately two weeks to remind them to complete and return the survey, or to arrange for the survey to be completed over the phone, via video-conferencing or in a face-to-face interview.

Table 1 includes an overview of **outcomes** (global health, function, cognition, QOL) and **needs**, and the time of collection.

**Table 1:** Overview of the data collected, instruments, and the time of collection

Data	Instrument	Source	Time of data collection		
			Baseline (TO)	3-months (T1)	6-months (T2)
**Participant characteristics**					
Date of birth	Questionnaire	Gatekeeper	●		
Sex	Questionnaire	Researcher	●		
Cohabiting	Questionnaire	Researcher	●		
Work status	Questionnaire	Researcher	●		
Home care -pre stroke	Questionnaire	Researcher	●		
**Disease-related data**					
Date of stroke	Questionnaire	Gatekeeper	●		
Type of stroke	Questionnaire	Gatekeeper	●		
Aphasia	Questionnaire	Gatekeeper	●		
Reperfusion therapy	Questionnaire	Gatekeeper	●		
Stroke severity (NIHSS)	Questionnaire	Gatekeeper	●		
**Outcomes**					
Global health	PROMIS 10	Researcher	●	●	●
Disability	smRS	Researcher	●	●	●
Cognition	T-MoCA	Researcher	●	●	●
Quality of life	EQ5D-5L	Researcher	●	●	●
**Needs**					
Needs	Stroke Survivor Needs Survey	Researcher	●	●	●

### Data analysis

All data were collated in, and analysed using Stata V16 [30]. Categorical data were coded and continuous data entered in numerical format. Appropriate descriptive statistics (e.g. means, standard deviations, frequencies, percentages) were used to report patient characteristics, levels and type of self-reported need, and outcome measures at each time point (Global Physical and Mental Health, Cognition, Function and QOL). All continuous variables were assessed for normality using histograms and Shapiro-Wilk tests. Means and standard deviations were calculated for normally distributed continuous variables, otherwise a median and interquartile range was calculated. Mixed effects models (logistic or linear regression as appropriate) were used to explore potential changes in outcomes of interest over time. All models accounted for the repeated measures over time, and were adjusted for age, sex and stroke severity. The models use all available data, even if some participants are missing data at one or two time points, thus all the available data points are used. Odds ratios or beta coefficients, 95% confidence intervals (CI) and p-values are reported.

Needs are presented in relation to physical and other stroke-related concerns, information, and support needs, Impact on social participation, active involvement in transition planning, and awareness of other support services. The total number of unmet or partially met needs that each respondent reported was determined by adding up the instances in which a need was described as “unmet” or “only partially met”. Responses to the opened-ended question on the needs survey were collated in excel [31], and analysed inductively. This involved an open, flexible and iterative process of reading and re-reading each response, following with line-by-line coding, leading to clustering and category generation, undertaken by GOC. Reflexive practice was facilitated through peer debriefing between GOC and supervisors (FH and RG). Through the discussion, we interpreted and synthesised the qualitative insights alongside the quantitative data to provide a comprehensive understanding of the research findings.

### Patient and public involvement (PPI)

Five “stroke champions” from the iPASTAR PPI panel, representing diverse demographics and experiences along the stroke pathway, worked collaboratively with the researcher to inform on study design and methods for this study (inclusion criteria, recruitment considerations, priority outcomes for data collection, methods of data collection), literature for ethics process (participant information leaflets, consent forms), pilot testing tools, and to interpret and discuss the findings. Collaborations and consensus building took place through meetings held on a video platform (MS Teams), and by e-mail. The Guidance for Reporting Involvement of Patient and Public version 2 short form (GRIPP2-SF) [32] is used to report on PPI in this review (supplemental material, Table II).

## Results

**Fig. 1:**
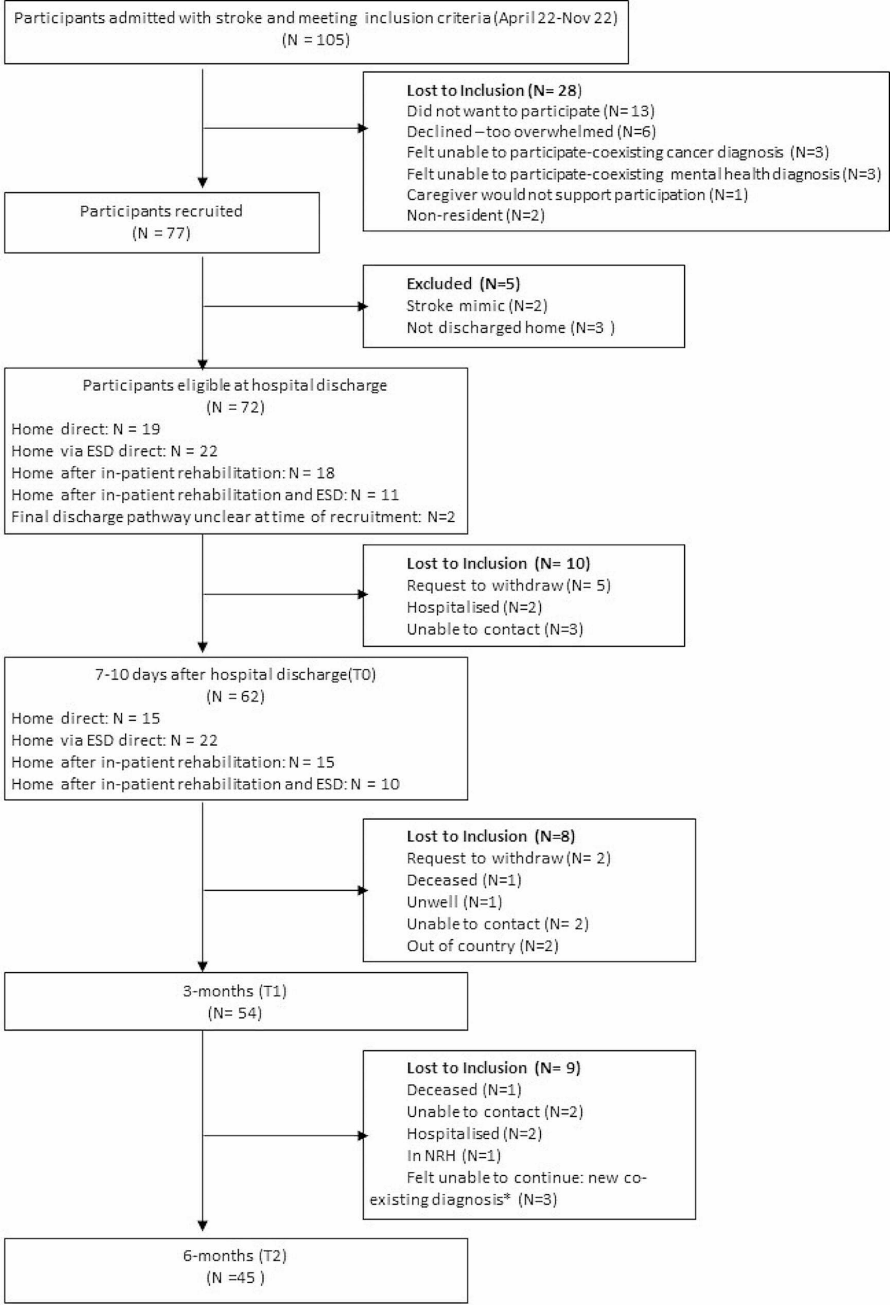
Flowchart of recruitment, inclusion and retention of people with stroke (*MND, Cancer, Liver failure)

Between March and December 2022, 105 participants met the inclusion criteria and were eligible to participate. Of those,77 people were enrolled in this study and 72 people were included in analysis. 45 participants completed the study. A flowchart outlining exclusion and retention of participants is contained in Fig. 1.

Participant characteristics and disease related data at time of discharge are presented in Table 2. Mean age was 70 (SD 13), 61% were female, median NIHSS was 4 (IQR 0–20). Of the 72 participants, 62 (86%) participants were available for outcome and needs evaluation at 7–10 days after hospital discharge (T0). At this time point five participants had requested to withdraw, two were hospitalised, and three were uncontactable. 54 (75%) participants were available at three months; and 45 (63%) were available at six months. Case fatality was 0 (0%) at-hospital-discharge, one (1.4%) at 3-months, and two (2.7%) at 6-months.

The characteristics of those lost to inclusion can be found in supplemental material, Table III. Upon analysing variables such as location, age, gender, type of stroke, stroke severity, and discharge home pathway, there were no statistically significant difference observed between those who completed the study and those who did not.

**Table 2:** Baseline characteristics, respondents eligible at hospital discharge, *n* = 72

Variable	Category	Number (percentage %)
**Location**	Site 1	24 (33)
	Site 2	28 (39)
	Site 3	20 (28)
**Age**	18–44	3 (4)
	45–64	19 (26)
	65–75	15 (21)
	75+	35 (49)
**Gender**	Male	44 (61)
	Female	28 (39)
**Type of stroke**	Ischemic	58 (81)
	Haemorrhage	14 (19)
**Thrombectomy**	Yes	9 (13)
	No	63 (87)
**Thrombolysis**	Yes	6 (8)
	No	66 (92)
**NIHSS (***n* **=** 59*)	0–4 Mild	31 (53)
	5–15 Moderate	22 (37)
	16–42 Severe	6 (10)
**Discharge Pathway (***n* **=** 70**)	Home direct	19 (27)
	Home via ESD	22 (31)
	Home via rehabilitation	18 (26)
	Home via rehabilitation + ESD^1^	11 (16)
**Length of Stay in Acute Hospital Setting (***n* **=** 71***)		Median 14 (IQR 4-105)

Participant data missing on *(*n* = 13), **(*n* = 2), ***(*n* = 1)

^1^ A cohort of participants transitioned from acute hospital to rehabilitation and were discharged home via ESD.

### Outcomes

Outcome Information is displayed in Table 3, more detailed information can be found in supplemental material Table IV.

At discharge to home PROMIS 10 mean t-score for physical health was 46.1 +/- 8.42, at three months was 45.51 +/- 8.1, and at six months was 46.5 +/- 7.63; while mean t-score for mental health at discharge to home was 46.97 +/- 10.98; at three months was 44.92 +/- 7, and at six months was 46.8 +/- 7.58 (Table 3). On average, there were small non-significant increases in PROMIS physical health over time (0.74 (-1.31–2.78), *p* = 0.48). There was decrease in PROMIS mental from T0 to T1 and an increase from T1 to T2, however these differences were not statistically significant (Table 4). Overall (from T0 to T2) there was an average decrease, but similarly this was not statistically significant (-0.56 (-3.25–2.13), *p* = 0.683). Information on individual domains, including social function, which was consistently rated over time as “fair”, can be found in supplemental material Table IV.

At hospital discharge 31 participants (51%) reported moderate to severe disability (mRS ≥ 3), 17 (32%) at three months, and 15 (35%) at six months. No participant reported severe disability at 6-months (Table 3). In regression analysis there was evidence of a lower odds of moderate to severe disability, compared to none to mild disability at T1 compared to T0 (OR: 0.05; 95% CI: 0.01 to 0.43) and also T2 compared to T0 (OR: 0.07; 95% CI: 0.01 to 0.54). Although there was an increase in odds at T2 compared to T1, this was not statistically significant (OR: 1.35; 95% CI: 0.22 to 8.18) (Table 4).

HR-QOL was impaired at hospital discharge (0.62 +/- 0.33), increasing to 0.68 +/- 0.24 at 3-months and 0.69 +/- 0.28 at 6-months (Table 3**)**. Regression analysis indicated an increase in EQ5D-5L at time 1 vs. time 0 (β = 0.07; 95%CI: 0.02 to 0.12), and similarly at time 2 vs. time 0 (β = 0.07; 95%CI: 0.02 to 0.12). There was no evidence of a change from time 1 to time 2, suggesting the increase occurred from T0 to T1 and remained stable from T1 to T2 (Table 4).

Cognitive impairment was observed in forty participants (68%) at discharge to home, and continued to present in seventeen participants (40%) at six months (Table 3**).** In regression analysis there was no evidence of a difference in the odds of cognitive impairment, compared to no cognitive impairment, at T1 compared to T0 (OR: 0.58; 95% CI: 0.16 to 2.06). There was however evidence of a difference seen at T2 compared to T0 (OR: 0.07; 95% CI: 0.01 to 0.38) and T2 compared to T1 (OR: 0.13; 95% CI: 0.03 to 0.60) (Table 4).

**Table 3:** Global Physical and Mental Health, Function, and Cognition at hospital discharge (T0), 3- (T1) and 6-months (T2) post-discharge

Global Physical and Mental Health (PROMIS 10 t-scores)	At hospital discharge (T0) (*n* = 61*)	At 3 months (T1) (*n* = 53*)	At 6 months (T2) (*n* = 45)
PROMIS physical (M +/- SD)	46.1 +/- 8.42	45.51 +/- 8.1	46.5 +/- 7.63
PROMIS mental (M +/- SD)	46.97 +/- 10.98	44.92 +/- 7	46.8 +/- 7.58
*Data were missing on one participant at T0 and T1 (< *2*%*).*			
**Functional outcome** **(smRS)**	**At hospital discharge (T0) (***n* **=** 61*)	**At 3 months** **(T1) (***n* **=** 53*)	**At 6 months** **(T2) (***n* **=** 44*)
None to mild disability (mRS 0–2) N(%)	30 (49)	36 (68)	28 (65)
Moderate disability (mRS 3–4) N(%)	28 (46)	15 (28)	15 (35)
Severe Disability** (mRS 5–6) N(%)	3 (5)	2 (4)	0 (0)
*Data were missing on one participant at T0, T1 and T2 (< *2*%*).* **Case fatality was 0 (0%) at-hospital-discharge, one (1.4%) at 3-months, and two (2.7%) at 6-months.			
**Health related quality of life (EQ5D-5L Index score)**	**At hospital discharge (T0) (***n* **=** 61*)	**At 3 months** **(T1) (***n* **=** 53*)	**At 6 months** **(T2) (***n* **=** **45)**
Index score (SD) (M +/- SD)	0.62 (0.33)	0.68 (0.24)	0.69 (0.28)
*Data were missing on one participant at T0 and T1 (< *2*%*).*			
**Cognitive Impairment MoCA (converted from T-MoCA)**	**At hospital discharge (T0) (***n* **=** 59 *)	**At 3 months (T1)** **(***n* **=** 48*)	**At 6 months (T2)** **(***n* **=** 43*)
No Cognitive Impairment (0–25) N(%)	19 (32)	19 (40)	26 (60)
Cognitive Impairment (26–30) N(%)	40 (68)	29 (60)	17 (40)

*Data were missing on 3 participants at T0 (5%*), 6 participants at T1 (11*%*), and 2 participants at T2 (4*%*).*

**Table 4:** Mixed effects regression analysis of Global Physical and Mental Health, Function, Health-related Quality of Life, and Cognition at hospital discharge (T0)

Global Physical and Mental Health (PROMIS 10 t-scores)	Number of participants*	Odds ratio (95% CI) P = value	Beta coefficient (95%CI) P = value
PROMIS physical	56		
Time 1 vs. Time 0			0.13 (-1.80 to 2.05) *p* = 0.897
Time 2 vs. Time 0			0.74 (-1.31 to 2.78) *p* = 0.480
Time 2 vs. Time 1			0.61 (-1.45 to 2.67) *p* = 0.563
PROMIS mental	56		
Time 1 vs. Time 0			-1.56 (-4.09 to 0.97) *p* = 0.227
Time 2 vs. Time 0			-0.56 (-3.25 to 2.13) *p* = 0.683
Time 2 vs. Time 1			1.00 (-1.72 to 3.71) *p* = 0.470
Functional outcome (smRS)**	56		
Time 1 vs. Time 0		0.05 (0.01 to 0.43) *p* = 0.006	
Time 2 vs. Time 0		0.07 (0.01 to 0.54) *p* = 0.011	
Time 2 vs. Time 1		1.35 (0.22 to 8.18) *p* = 0.743	
**Health related quality of life (EQ5D-5L Index score)**	56		
Time 1 vs. Time 0			0.07 (0.02 to 0.12) *p* = 0.010
Time 2 vs. Time 0			0.07 (0.02 to 0.12) *p* = 0.011
Time 2 vs. Time 1			0.004 (-0.05 to 0.06) *p* = 0.895
Cognitive Impairment MoCA (converted from T-MoCA)***	54		
Time 1 vs. Time 0		0.58 (0.16 to 2.06) *p* = 0.400	
Time 2 vs. Time 0		0.07 (0.01 to 0.38) *p* = 0.002	
Time 2 vs. Time 1		0.13 (0.03 to 0.60) *p* = 0.009	

All models are adjusted for age, sex and stroke severity.

*With data available for at least one time point.

** Binary variable: Moderate/Severe disability defined as mRS 3–6 vs. None to mild disability defined as an mRS 0–2.

***Binary variable: No cognitive impairment (0–25) vs. Cognitive impairment (26–30)

### Needs

Needs Information is displayed in Tables 4 and 5, more detailed information can be found in supplemental material Tables Va–Vc, and VI to IX.

At hospital discharge seven participants (12%) reported having no unmet health needs; among the remaining participants, the median number of unmet needs was 4 (range: 1–9), including emotional issues (49%), memory (44%), fatigue (37%), and communication (35%). At 6-months, the median number of unmet needs was 3 (range: 1–9), with memory (38%), emotional issues (29%), pain (25%) and fatigue (24%) the leading unmet health needs (Table 5).

At hospital discharge over 80% of people with support and information needs primarily required information regarding their stroke, followed by nutritional advice (51%), assistance with driving (40%), and financial support (32%) (supplemental material Table VI). These information and support needs persisted at 3- and 6-months.

On discharge 51% of participants did not have an opportunity to discuss their transition to home plan with their healthcare team, and 36% stated that they were not involved in decisions about their care and treatment (supplemental material Table VIII). At this point only two participants (4%) were unaware of stroke support groups, compared to thirteen (22%) at discharge to home (supplemental material Table IX).

**Table 5:** Proportion of respondents reporting a stroke-related problem at hospital discharge (T0), 3- (T1) and 6-months (T2) post-discharge, and the extent to which needs are unmet

Issue with	T0- (*n* = 59)*		T1- (*n* = 54)*		T2- (*n* = 45)*	
	No. reporting an issue (%)	No reporting need unmet (%)	No. reporting an issue (%)	No reporting need unmet (%)	No. reporting an issue (%)	No reporting need unmet (%)
**Mobility**	53 (90)	13 (25)	46 (85)	11 (24)	40 (89)	9 (22)
**Falls**	50 (85)	14 (28)	40 (74)	11 (28)	37 (82)	7 (19)
**Continence**	25 (43)	6 (24)	22 (41)	4 (19)	16 (36)	3 (18)
**Pain**	28 (47)	7 (25)	26 (48)	5 (19)	24 (53)	6 (25)
**Fatigue**	43 (73)	16 (37)	40 (74)	11 (28)	29 (64)	7 (24)
**Emotion**	39 (66)	19 (49)	36 (67)	14 (39)	28 (62)	8 (29)
**Concentration**	25 (42)	5 (20)	24 (44)	6 (25)	20 (44)	2 (10)
**Memory**	27 (46)	12 (44)	26 (48)	12 (46)	21 (47)	8 (38)
**Speech**	23 (39)	8 (35)	22 (41)	8 (36)	18 (40)	4 (22)
**Reading**	21 (36)	6 (29)	17 (31)	4 (24)	14 (31)	0 (0)
**Sight**	26 (42)	8 (31)	24 (44)	6 (25)	20 (44)	3 (15)
**Personal care**	25 (42)	4 (16)	21 (39)	1 (5)	19 (42)	1 (5)
**Home help**	20 (32)	9 (45)	16 (30)	8 (50)	15 (33)	6 (40)

*Data was missing on 13 participants at T0, 18 participants at T1, and 27 participants at T2

Finally, a number of key categories emerged during an analysis of the open-ended needs survey question that asked about other requirements not covered by the survey questions (Table 6). Coding, category generation and a narrative overview can be found in supplemental material Table X.

**Table 6:** Generation of categories from open-ended question on Stroke Survivors Needs Survey asking about other requirements not covered by the survey questions

Category	Unmet-need
Processes for Successful Transition to Home and Life After Stroke	**“**Disabled persons housing grant is too slow, and people are struggling unnecessarily because it’s so slow”
Empowering People with Stroke and Families through Comprehensive Health and Social Care Information	**“**Can’t take everything in in hospital and now there is no information available”
Navigating Stroke Journeys Together	**“**Concrete plan with goal setting and targets. This needs to be a partnership, identify the problems together, and solve problems together”
Effective Communication and Information Sharing for Individualised Healthcare	“Blood pressure medications now lower than what I was previously on and my GP didn’t know I had a stroke”
Comprehensive Whole-Systems Approach to Rehabilitation and Recovery	**“**Information on the Irish Heart Foundation* - heard rumours of what’s available but nothing concrete”
Empowering Families	**“**Family involved in getting information about stroke - especially when it is clear the patient does not understand the information”
Keyworker / Dedicated Case Manager Role: a Bridge between Healthcare Settings and Person with Stroke / Families	**“**Link person between the acute and community to field questions”
Comprehensive Monitoring and Support for Residual Needs and Long-Term Stroke Recovery	**“**Follow-up after 3-months (by ESD team), for review and residual needs assessment and signposting as appropriate - “to do a final signoff”, to be able to ask questions about symptoms that emerge in long-term, to help adjust and accept any mild deficits. This would help you to accept the stroke”

GP = General Practitioner; *The Irish Heart Foundation is a registered charity, who play a significant role in supporting individuals and families in Ireland affected by stroke and heart disease

## Discussion

This is the first Irish prospective cohort study to profile outcomes and document levels of unmet need in people with stroke at transition to home, and up to 6-months follow-up.

The study found that 51% of participants experienced moderate to severe disability at hospital discharge, with improvement in the first three months but a deterioration by six months. Further analysis, though not statistically significant, suggested this trend towards increasing disability between T1 and T2. Our study observed significant improvements in health-related quality of life (HRQOL) from hospital discharge to the six-month follow-up period, however, the majority of these improvements occurred within the first three months. While not statistically significant, there was evidence indicating a decline in mental health from baseline to the six-month mark. Although regression analysis indicated a decrease in the odds of cognitive impairment over time, the most significant finding underscores the persistent prevalence of cognitive impairment at six months (40%).

This study also highlights the range of unmet needs of individuals with stroke during transition to home. One notable finding is the number of missed opportunities for individuals to engage in discussions about their home plans and to participate in care decisions, highlighting a significant gap in patient-centred care during the transition process. Unmet rehabilitative needs included, but were not limited to, emotional issues, memory, fatigue, and communication. Individuals with stroke require tailored information and education about stroke and recovery, as well as support around nutrition, driving and finances.

In light of the knowledge that substantial functional gains are possible in both the short and long-term after stroke [33, 34], our data, which points to a shift in the direction of increasing disability between three and six months after stroke, warrants further investigation. Our findings are comparable with a large longitudinal study which indicated functional dependence increases from 3-months (16%) to 1-year (28%) [6], however; our study reported a higher proportion of those with moderate to severe disability at 6-months (35%) compared to López-Espuela et al. where only 13.3% were moderately to severely disabled at this juncture [35]. Among the factors associated with decreased functional status at 6 months were stroke severity, mood and social risk (isolation and social support) [35].

While significant improvements were noted over time in health-related quality of life (HRQOL), this plateaued from 3 to 6-months. There was also evidence indicating a decline in mental health between baseline and six-months. A large German study found that HR-QOL is impaired after stroke, and reported findings of mental health at three months similar to our study’s findings (44.3 +/- 8.63 at 3-months) [5]. A plateau in perceived quality of life and evidence of deteriorating mental health may be explained by the person’s initial excitement on returning home after stroke, which wanes as the realities of adjusting and coping with post-stroke changes become apparent. However, these changes could also be attributed to the ongoing presence of cognitive impairment and reduced social function found in this study, which is consistent with research discussing the impact of these factors on QOL [36], and function [35]. A study in China found participation self-efficacy to be a major factor associated with post stroke depression [37]. This study highlights the significance of monitoring for cognition, mental health and quality of life over the transition period, and indicate that early-targeted interventions post-discharge may be critical to improve these outcomes.

International evidence supports our finding of high levels of residual and ongoing unmet needs after stroke [8]. Although we identified three as the median number of unmet needs at 6-months post-hospital discharge, this number persisted as the median number of unmet needs > 1 year after stroke [4]. A Swedish study reported 21% of its participants experienced unmet needs during the same period [38]. Similar to our findings, studies identify memory, fatigue, concentration, emotional problems, and mobility as highest in terms of unmet rehabilitation needs [4, 9], with emotional challenges persisting even when formal transition to home support systems such as ESD are in place [12]. People with stroke and service providers have identified emotional well-being as a top long-term priority after stroke; impacted by the individuals perseverance and adaptability, peer support, and timely and appropriate community-based professional support [39, 40]. These findings underscore the importance of prioritising emotional health as a fundamental aspect of long-term rehabilitation, developing targeted interventions that might include goal setting and motivational strategies, connecting individuals with support networks, and improving access to rehabilitation.

When responding to the open-ended question regarding additional needs, people with stroke identified a need for improved communication and information-sharing processes between healthcare providers and individuals with stroke and their families at transition to home. Information-sharing is crucial for seamless care transitions, improving efficiency and patient experience [41], however, the evidence for the effectiveness of information delivered at the transition to home is variable [14]. More recently it has been determined that information which is tailored to the individual, delivered in mixed formats and at multiple time-points, and with the person with stroke as an active participant, can impact post-stroke anxiety and patient satisfaction [42, 43]. Participants identified a need for a dedicated keyworker/case manager to facilitate a more seamless transition. Previous studies found that a case manager role can improve depression, and adherence to self-care practices, and some aspects of QOL [44, 45], but not in isolation [45]; while another found no effect on function, QOL, mood or healthcare utilisation [46]. Calls for a dedicated keyworker/case manager as part of standardised transition to home planning have been echoed by members of the Movement Interventions Task Force in America [47], who recommend the transition specialist is a “receiving provider” situated among community based health staff, able to disseminate information to community colleagues, and support patient engagement and access to resources [47]. Depending on the role definition, a transition specialist integrated into the stroke pathway may be a catalyst for a whole systems approach to rehabilitation and recovery.

While rehabilitation services and health education are primary needs at transition home after stroke [48], our study documents a wider range of multi-dimensional unmet needs after stroke, including clinical and social care domains, services, information and accessibility, and social participation including leisure, driving and employment, which impact on the individual, society, and the economy. Findings highlight the need for an individualised, comprehensive approach to stroke care that monitors and takes into account the many needs of stroke survivors in different contexts as they adjust to living at home. This study supports recommendations from a Delphi process which determines an integrated pathway and whole-systems approach to rehabilitation and recovery is required, one which engages collaboration and cooperation across health, social care, and voluntary and community sectors [41].

### Strengths and limitations

This prospective cohort study is among a limited number to consider changes in unmet need over time. Its contributions are important in understanding the unmet needs of people with stroke as they transition to home. However the findings need to be interpreted with caution in the context of a number of study limitations. Concerns about precision in selected outcome measures may arise. PROMIS 10 is recommended for stroke research due to its ability to assess multiple stroke domains, including fatigue and emotional issues [24]; its moderate internal reliability, convergent validity, and excellent discriminant validity across mRS [25]; however, it’s adoption is limited and studies allowing comparison are scarce; and it may be more appropriate in detecting differences in those with mild to moderate stroke [49]. We recruited a small sample across the three sites, a predominance of participants were over 75 years, and with mild-to-moderate stroke. While every effort was made to enrol consecutive participants, this may not always have been possible due to work-flow issues on participating sites. These limitations may challenge the generalisability of study findings. Additionally, self-reported data collection, subjective and potentially unrelated to stroke, can lead to bias in under- or over-rating abilities or needs. It should however be noted, however, that Irish respondents to health questionnaires have a tendency to over-rate their health [50], therefore policy makers and healthcare providers should acknowledge this, and be responsive to reported needs within this study. Finally, while our needs survey incorporated an open-ended question to capture perspectives of PWS during the transition to home, there are limitations in the depth and breadth of data gathered; future research by this group will address this by incorporating other qualitative research methods to comprehensively delve into the nuanced experiences and perspectives of PWS.

### Policy and practice implications

Implementing a needs assessment at periodic intervals after stroke provides useful insights on the practice and policy implications of following recommendations for a comprehensive, organised needs reassessment be undertaken at 6-months [16, 17]. Clinicians and policy makers should include a periodic needs assessment, at discharge-to-home and at 6-months, into current systems and guidelines, to ensure potential issues are identified early, allowing for more efficient resource allocation and targeted interventions.

### Future research

Alongside the stated benefits of including a needs assessment at discharge-to-home, we believe an open-ended question, which asks about additional needs, adds to the richness of data collected, and better allows participants to identify and articulate their individualised needs. Further research is required to develop a robust framework and appropriate tool for monitoring and evaluating needs, and to review the feasibility of these recommendations in practice.

While uncertainty exists around interventions that effectively support the needs of individuals at transition to home and in the first six months after stroke [14], focus is growing on co-developing transition-to-home research [51], supporting suggestions that experienced-based approaches to intervention development can facilitate the emergence of services that are tailored to the individual and better meet the needs of a target population and contribute to better outcomes [52, 53]. Future research should engage people with stroke, alongside caregivers and healthcare professionals in a co-design process to develop an intervention to support the transition from hospital to home after stroke.

### Patient and public involvement (PPI)

The lived experience of the iPASTAR PPI contributors in this study allowed them to engage throughout the design, data analysis and interpretation phases of the study. This level of participation enabled us to develop a robust study, discuss aspects pertinent to the study’s intended users, and to establish future research priorities. Next steps are to establish a dissemination strategy collaboratively, that engages PPI in co-dissemination of the research.

## Conclusion

Stroke community reintegration is challenging for people with stroke. This study offers robust estimates of the outcomes and levels of unmet need of people with stroke in Ireland, providing valuable insights into resource gaps at transition to home and life after stroke. It recommends health services adopt needs assessments at transition to home, and 6-months after stroke, to enhance the effectiveness of their decision-making processes and promote more targeted and impactful interventions to address the evolving needs of people with stroke.

### Electronic supplementary material

Below is the link to the electronic supplementary material.

Supplementary Material 1

## Data Availability

The dataset used and analysed during the current study are available from the corresponding author upon reasonable request.
