# Supplementary material for: Transitioning to home and beyond following stroke: a prospective cohort study of outcomes and needs
Source: BMC Health Serv Res. 2024 Apr 10;24:449. doi: 10.1186/s12913-024-10820-8 (PMC11005232; doi:10.1186/s12913-024-10820-8)
Supplement: Supplementary file 1 — Supplementary Material 1 [file 12913_2024_10820_MOESM1_ESM.docx]

**SUPPLEMENTAL MATERIAL**

**for**

**Transitioning to home and beyond following stroke: a prospective cohort study of outcomes and needs.**

**Authors and Affiliations:**

Geraldine O’Callaghan^1^, Martin Fahy^2^, Sigrid O’Meara^2^, Mairead Chawke^3^, Eithne Waldron^3^, Marie Corry^3^, Sinead Gallagher^4^ , Catriona Coyne^4^ , Julie Lynch^5^, Emma Kennedy^5^ , Dr Thomas Walsh^6^, Dr Hilary Cronin^7^, Dr Niamh Hannon^8^, Prof Clare Fallon^9^, Prof David J Williams^10^, Prof Peter Langhorne^11^, Prof Rose Galvin^12^, Prof Frances Horgan^1^

^1.^iPASTAR Collaborative Doctoral Award Programme, School of Physiotherapy, RCSI University of Medicine and Health Sciences, 123 St. Stephen's Green, Dublin 2, Ireland

^2.^PPI Champion, iPASTAR Collaborative Doctoral Award Programme, RCSI Division of Population Health Sciences, RCSI University of Medicine and Health Sciences, 123 St. Stephen's Green, Dublin 2, Ireland

^3.^Early Supported Discharge Team for Stroke, Galway University Hospital, Newcastle Rd, Galway, H91 YR71

^4.^Acute Stroke Team, Regional Hospital Mullingar, Co. Westmeath, Ireland N91 NA43

^5.^Acute Stroke Team, Beaumont Hospital, Dublin 9, Ireland D09V2N0

^6.^ Consultant Geriatrician / Stroke Physician, Stroke and Geriatric Medicine, Galway University Hospital, Newcastle Rd, Galway, Ireland. H91 YR71

^7.^ Consultant Geriatrician, Regional Hospital Mullingar, Co. Westmeath, Ireland. N91 NA43

^8.^ Consultant Stroke Physician, Stroke and Geriatric Medicine, Galway University Hospital, Newcastle Rd, Galway, Ireland. H91 YR71

^9.^ Consultant Geriatrician, General Internal Medicine Physician & RCSI Undergraduate Dean, Regional Hospital Mullingar, Co. Westmeath, Ireland. N91 NA43

^10.^ Department of Geriatric and Stroke Medicine and iPASTAR Collaborative Doctoral Award Programme, RSCI University of Medicine and Health Sciences and Beaumont Hospital, Dublin 9, Ireland

^11.^ Senior Research Fellow, School of Cardiovascular and Metabolic Health (SCMH), University of Glasgow, Scotland.

^12.^ School of Allied Health, Faculty of Education and Health Sciences, Ageing Research Centre, Health Research Institute, University of Limerick, Limerick, Ireland

Corresponding author: Geraldine O’Callaghan. iPASTAR Collaborative Doctoral Award Programme, RCSI School of Physiotherapy, RCSI University of Medicine and Health Sciences, 123 St. Stephen's Green, Dublin 2, Ireland

Email: gocallaghan@rcsi.com

**Supplemental information included**:

Table I: STROBE Checklist V4

Table II: GRIPP2 short form

Table III: PROMIS 10 Subdomains

Table IV: Total recruited, total loss to attrition, and total completing the study, by variable.

Table Va: Proportion of respondents reporting stroke-related unmet needs at T0, and the extent to which needs are met

Table Vb: Proportion of respondents reporting stroke-related unmet needs at T1, and the extent to which needs are met.

Table Vc: Proportion of respondents reporting stroke-related unmet needs at T2, and the extent to which needs are met.

Table VI: Proportion of respondents reporting need for information / support at hospital discharge (T0), 3- (T1) and 6-months (T2) post-discharge

Table VII: Reported change since stroke

Table VIII: Involved in Transition Planning

Table IX: Attendance at support group

Table X: Codes, categories and Higher Order categories at T0; T1, and T2, plus narrative summary

**Table I:** STROBE Statement—Checklist of items that should be included in reports of ***cohort studies***

|  | Item No | Recommendation |
| --- | --- | --- |
| **Title and abstract** | 1 | (*a*) Indicate the study’s design with a commonly used term in the title or the abstract |
|  |  | (*b*) Provide in the abstract an informative and balanced summary of what was done and what was found |
| Introduction | | |
| Background/rationale | 2 | Explain the scientific background and rationale for the investigation being reported |
| Objectives | 3 | State specific objectives, including any pre-specified hypotheses |
| Methods | | |
| Study design | 4 | Present key elements of study design early in the paper |
| Setting | 5 | Describe the setting, locations, and relevant dates, including periods of recruitment, exposure, follow-up, and data collection |
| Participants | 6 | (*a*) Give the eligibility criteria, and the sources and methods of selection of participants. Describe methods of follow-up |
|  |  | (*b*) For matched studies, give matching criteria and number of exposed and unexposed |
| Variables | 7 | Clearly define all outcomes, exposures, predictors, potential confounders, and effect modifiers. Give diagnostic criteria, if applicable |
| Data sources/ measurement | 8* | For each variable of interest, give sources of data and details of methods of assessment (measurement). Describe comparability of assessment methods if there is more than one group |
| Bias | 9 | Describe any efforts to address potential sources of bias |
| Study size | 10 | Explain how the study size was arrived at |
| Quantitative variables | 11 | Explain how quantitative variables were handled in the analyses. If applicable, describe which groupings were chosen and why |
| Statistical methods | 12 | (*a*) Describe all statistical methods, including those used to control for confounding |
|  |  | (*b*) Describe any methods used to examine subgroups and interactions |
|  |  | (*c*) Explain how missing data were addressed |
|  |  | (*d*) If applicable, explain how loss to follow-up was addressed |
|  |  | (*e*) Describe any sensitivity analyses |
| Results | | |
| Participants | 13* | (a) Report numbers of individuals at each stage of study—eg numbers potentially eligible, examined for eligibility, confirmed eligible, included in the study, completing follow-up, and analysed |
|  |  | (b) Give reasons for non-participation at each stage |
|  |  | (c) Consider use of a flow diagram |
| Descriptive data | 14* | (a) Give characteristics of study participants (eg demographic, clinical, social) and information on exposures and potential confounders |
|  |  | (b) Indicate number of participants with missing data for each variable of interest |
|  |  | (c) Summarise follow-up time (eg, average and total amount) |
| Outcome data | 15* | Report numbers of outcome events or summary measures over time |
| Main results | 16 | (*a*) Give unadjusted estimates and, if applicable, confounder-adjusted estimates and their precision (eg, 95% confidence interval). Make clear which confounders were adjusted for and why they were included |
|  |  | (*b*) Report category boundaries when continuous variables were categorized |
|  |  | (*c*) If relevant, consider translating estimates of relative risk into absolute risk for a meaningful time period |
| Other analyses | 17 | Report other analyses done—eg analyses of subgroups and interactions, and sensitivity analyses |
| Discussion | | |
| Key results | 18 | Summarise key results with reference to study objectives |
| Limitations | 19 | Discuss limitations of the study, taking into account sources of potential bias or imprecision. Discuss both direction and magnitude of any potential bias |
| Interpretation | 20 | Give a cautious overall interpretation of results considering objectives, limitations, multiplicity of analyses, results from similar studies, and other relevant evidence |
| Generalisability | 21 | Discuss the generalisability (external validity) of the study results |
| Other information | | |
| Funding | 22 | Give the source of funding and the role of the funders for the present study and, if applicable, for the original study on which the present article is based |

*Give information separately for exposed and unexposed groups.

**Table II:** GRIPP2 short form

| **Section and topic** | **Item** | **Reported on page No** |
| --- | --- | --- |
| **1: Aim** | Report the aim of PPI in the study | 2 |
| **2: Methods** | Provide a clear description of the methods used for PPI in the study | 6 |
| **3: Study results** | Outcomes—Report the results of PPI in the study, including both positive and negative outcomes | Supplemental material |
| **4: Discussion and conclusions** | Outcomes—Comment on the extent to which PPI influenced the study overall. Describe positive and negative effects | 19  &  Supplemental material |
| **5: Reflections/critical perspective** | Comment critically on the study, reflecting on the things that went well and those that did not, so others can learn from this experience | Supplemental material |

Outcomes / Discussion / Reflections.

iPASTAR PPI contributors were invaluable sources of information, influence and support during the course of this study. Their lived experience of stroke enabled them to be involved throughout the preliminary stage, design, data analysis and interpretation, and dissemination.

By engaging with PPI and other stakeholders we co-developed the research question; evaluated the outcomes that are priorities for stroke survivors and caregivers and reflected this in reporting; and guaranteed that findings were discussed in a way that considers what is relevant and meaningful to people affected by stroke.

In response to PPI feedback we were prepared for potential recruitment challenges, and were assured that outcomes collected are a priority for people with stroke. We included flexibility in methods of data collection which impacted on recruitment and retention rates, and having the ability to pilot test the forms reduced potential participant burden. PPI contributors offered insights into findings and identified aspects for discussion that would be of relevance to the intended users of the review. This level of engagement allowed us to identify future research priorities, and further collaboration will inform a dissemination strategy.

iPASTAR PPI contributors shared their perspectives on the research and highlighted topics for debate that would be relevant to the review's target audience. Continued partnership will inform a dissemination strategy.

**Table III:** PROMIS-10 Subdomains**:**

|  | **T0 (n=61*)**  **M (SD)** | **T1 (n=53*)**  **M (SD)** | **T2 (n=45*)**  **M (SD)** |
| --- | --- | --- | --- |
| **PROMIS_rate general health** | 3.05 (1.07) | 3.09 (0.90) | 3.15 (0.82) |
| **PROMis_rate quality of life** | 3.32 (1.18) | 2.92 (0.82) | 3.17 (0.93) |
| **PROMIS_rate physical health** | 2.93 (1.12) | 2.69 (1.06) | 2.82 (0.83) |
| **PROMIS_rate mental health** | 3.47 (0.97) | 3.07 (0.95) | 3.31 (0.94) |
| **PROMIS_rate satisfaction with social activities and relationships** | 3.32 (1.31) | 3.01 (1.06) | 3.22 (0.95) |
| **PROMIS_ social function** | 2.66 (1.28) | 2.84 (1.04) | 2.68 (1.10) |
| **PROMIS_carry out every day physical activities** | 3.26 (1.28) | 3.43 (1.20) | 3.42 (1.17) |
| **PROMIS_bothered by emotional problems** | 3.45 (0.97) | 3.43 (1.20) | 3.64 (1.00) |
| **PROMIS_rate fatigue** | 3.44 (0.95) | 3.52 (1.03) | 3.71 (0.96) |
| **PROMIS_rate pain** | 4.27 (1.03) | 4.30 (0.91) | 4.44 (0.84) |

**Table IV**. Total recruited, total loss to attrition, and total completing the study, by variable.

|  |  | **Total recruited**  **N=72** | **Total loss to attrition from recruitment to T2 (n=27)** | **Total completing study (n=45)** | **P value*** |
| --- | --- | --- | --- | --- | --- |
| **Variable** | **Category** | **Recruited in each category** | **N**  **(%)** | **N**  **(%)** |  |
| **Location** | Site 1 | 24 (33) | 8 (30) | 17(38) | 0.885 |
|  | Site 2 | 28 (39) | 13 (48) | 15(33) |  |
|  | Site 3 | 20 (28) | 6(22) | 13(29) |  |
|  |  |  |  |  |  |
| **Age** | 18-44 | 3 (4) | 2 (7) | 2(4) | 0.357 |
|  | 45-64 | 19 (26) | 11 (41) | 8(18) |  |
|  | 65-75 | 15 (21) | 4 (15) | 11(25) |  |
|  | 75+ | 35 (49) | 10 (37) | 24(53) |  |
|  |  |  |  |  |  |
| **Gender** | Male | 44 (61) | 18 (67) | 26(58) | 0.422 |
|  | Female | 28 (39) | 9 (33) | 19(42) |  |
|  |  |  |  |  |  |
| **Type of stroke** | Ischemic | 58 (81) | 20 (74) | 37(82) | 0.251 |
|  | Haemorrhage | 14 (19) | 7 (26) | 8(18) |  |
|  |  |  |  |  |  |
| **Stroke severity**  **(NIHSS)**** | 0-4 Mild | 31 (53) | 9(43) | 23(56) | 0.788 |
|  | 5-15 Moderate | 22 (37) | 9(43) | 14(34) |  |
|  | 16-42 Severe | 6 (10) | 3(14) | 4(10) |  |
|  |  |  |  |  |  |
| **Discharge pathway***** | Home direct | 20(28) | 7(31) | 11(24) | 0.992 |
|  | Home via ESD | 21(30) | 7(31) | 14(32) |  |
|  | Home after inpatient rehabilitation | 18(26) | 7(31) | 11(24) |  |
|  | Home after inpatient rehabilitation and ESD | 11(16) | 2(7) | 9(20) |  |

*Chi^2^ or fishers exact where appropriate

**NIHSS data missing on 13 participants at T0, on 6 who were lost to attrition, and on 4 who completed the study.

***Discharge pathway missing on 6 participants at T0, and on 4 who were lost to attrition.

**Table Va**. Proportion of respondents reporting **stroke-related unmet needs** at T0, and the extent to which needs are met.

| **T0- Issues with**  **(n=59)*** | No. reporting an issue (%) | No. reporting need unmet (%) | No. reporting need met to some extent (%) | No. reporting need met (%) | No. not wanting support (%) |
| --- | --- | --- | --- | --- | --- |
| Mobility | 53 (90) | 13 (25) | 9 (17) | 31 (58) | 0 (0) |
| Falls | 50 (85) | 14 (28) | 8 (16) | 28 (56) | 0 (0) |
| Continence | 25 (43) | 6 (24) | 2 (8) | 13 (52) | 4 (16) |
| Pain | 28 (47) | 7 (25) | 7 (25) | 14 (50) | 0 (0) |
| Fatigue | 43 (73) | 16 (37) | 8 (19) | 9 (21) | 10 (23) |
| Emotion | 39 (66) | 19 (49) | 8 (21) | 6 (15) | 6 (15) |
| Concentration | 25 (42) | 5 (20) | 6 (24) | 9 (36) | 5 (20) |
| Memory | 27 (46) | 12 (44) | 7 (26) | 7 (26) | 1 (4) |
| Speech | 23 (39) | 8 (35) | 6 (26) | 7 (30) | 2 (9) |
| Reading | 21 (36) | 6 (29) | 4 (19) | 5 (23) | 6 (29) |
| Sight | 26 (42) | 8 (31) | 9 (34) | 4 (15) | 4 (15) |
| Personal care | 25 (42) | 4 (16) | 5 (20) | 16 (64) | - |
| Home help | 20 (32) | 9 (45) | 3 (15) | 8 (40) | - |

**Table Vb**. Proportion of respondents reporting **stroke-related unmet needs** at **T1**, and the extent to which needs are met.

| **T1- Issues with**  **(n=54)** | No. reporting an issue (%) | No. reporting need unmet (%) | No. reporting need met to some extent (%) | No. reporting need met (%) | No. not wanting support (%) |
| --- | --- | --- | --- | --- | --- |
| Mobility | 46 (85) | 11 (24) | 12 (26) | 26 (50) | 0 (0) |
| Falls | 40 (74) | 11 (28) | 10 (25) | 17 (42) | 2 (5) |
| Continence | 22 (41) | 4 (19) | 6 (27) | 6 (27) | 6 (27) |
| Pain | 26 (48) | 5 (19) | 6 (23) | 6 (23) | 9 (35) |
| Fatigue | 40 (74) | 11 (28) | 7 (17) | 9 (23) | 13 (32) |
| Emotion | 36 (67) | 14 (39) | 2 (6) | 4 (11) | 16 (44) |
| Concentration | 24 (44) | 6 (25) | 3 (13) | 4 (16) | 11 (46) |
| Memory | 26 (48) | 12 (46) | 5 (19) | 3 (12) | 6 (23) |
| Speech | 22 (41) | 8 (36) | 4 (18) | 6 (27) | 4 (18) |
| Reading | 17 (31) | 4 (24) | 3 (18) | 4 (24) | 6 (35) |
| Sight | 24 (44) | 6 (25) | 5 (21) | 5 (21) | 8 (33) |
| Personal care | 21 (39) | 1 (5) | 3 (14) | 17 (81) | - |
| Home help | 16 (30) | 8 (50) | 2 (13) | 6 (37) | - |

**Table Vc**. Proportion of respondents reporting **stroke-related unmet needs** at **T2**, and the extent to which needs are met.

| **T2- Issues with**  **(n=45)** | No. reporting an issue (%) | No. reporting need unmet (%) | No. reporting need met to some extent (%) | No. reporting need met (%) | No. not wanting support (%) |
| --- | --- | --- | --- | --- | --- |
| Mobility | 40 (89) | 9 (22) | 12 (30) | 17 (43) | 2 (5) |
| Falls | 37 (82) | 7 (19) | 10 (27) | 19 (51) | 1 (3) |
| Continence | 16 (36) | 3 (18) | 3 (18) | 6 (38) | 4 (6) |
| Pain | 24 (53) | 6 (25) | 5 (20) | 9 (38) | 4 (17) |
| Fatigue | 29 (64) | 7 (24) | 6 (21) | 6 (21) | 10 (34) |
| Emotion | 28 (62) | 8 (29) | 6 (21) | 7 (25) | 7 (25) |
| Concentration | 20 (44) | 2 (10) | 1 (5) | 10 (50) | 7 (35) |
| Memory | 21 (47) | 8 (38) | 2 (10) | 6 (28) | 5 (24) |
| Speech | 18 (40) | 4 (22) | 2 (11) | 9 (50) | 3 (17) |
| Reading | 14 (31) | 0 (0) | 2 (14) | 7 (50) | 5 (36) |
| Sight | 20 (44) | 3 (15) | 2 (10) | 9 (45) | 6 (30) |
| Personal care | 19 (42) | 1 (5) | 1 (5) | 17 (90) | - |
| Home help | 15 (33) | 6 (40) | 0 (0) | 9 (60) | - |

**Table VI.** Proportion of respondents reporting need for information / support at hospital discharge (T0), 3- (T1) and 6-months (T2) post-discharge

| **Need for information / support relating to** | **T0 (n=59)***  **No. of respondents (%)** | **T1 (n=54)**  **No. of respondents**  **(%)** | **T2 (n=45)**  **No. of respondents**  **(%)** |
| --- | --- | --- | --- |
| Stroke | 47 (80) | 42 (78) | 30 (67) |
| Diet | 30 (51) | 23 (43) | 13 (29) |
| Home Aids | 17 (29) | 10 (19) | 6 (13) |
| Home Adaptations | 9 (15) | 6 (11) | 5 (11) |
| Moving home | 2 (3) | 3 (6) | 4 (9) |
| Driving / permit | 23 (40) | 19 (35) | 11 (24) |
| Public transport | 4 (7) | 8 (15) | 4 (9) |
| Holidays | 13 (22) | 14 (26) | 6 (13) |
| Sexual relations | 3 (5) | 2 (4) | 1 (2) |
| Benefits | 19 (32) | 16 (30) | 9 (20) |
| Money management | 4 (7) | 4 (7) | 3 (7) |
| Employment | 7 (12) | 5 (9) | 5 (11) |

*Data was missing on 13 participants at T0, 18 participants at T1, and 27 participants at T2.

**Table VII**. Reported change since stroke

| **Change since stroke reported in** | **T0 (n=59)**  **No. of respondents (%)** | **T1 (n=54)**  **No. of respondents (%)** | **T1 (n=45)**  **No. of respondents (%)** |
| --- | --- | --- | --- |
| Transport / travel | 43 (73) | 29 (54) | 21 (47) |
| Work | 19 (32) | 12 (22) | 8 (18) |
| Leisure | 32 (55) | 27 (50) | 19 (42) |
| Relationships – partner/spouse | 12 (20) | 9 (16) | 8 (18) |
| Relationships – family | 9 (15) | 9 (17) | 8 (18) |
| Relationships – other | 8 (14) | 16 (30) | 7 (16) |
| Income | 12 (20) | 8 (15) | 5 (11) |
| Increased expenses | 22 (37) | 23 (43) | 16 (36) |

**Table VIII:** Involved in Transition Planning**.**

| **Transition planning** | **T0 (n=59)**  **No. of respondents**  **Yes (%)** | **T1 (54)**  **No. of respondents**  **Yes (%)** | **T1 (45)**  **No. of respondents**  **Yes (%)** |
| --- | --- | --- | --- |
| Opportunity to discuss transition plan | 29 (49) |  |  |
| Had Blood pressure checked in last 3mths |  | 40 (74) | 33 (73) |
| Involved in decisions about care & treatment | 38 (64) | 36 (67) | 32 (71) |

**Table IX:** Attendance at support group

| **Stroke support groups** | **T0 (n=59)**  **No. of respondents Yes (%)** | **T1 (54)**  **No. of respondents**  **Yes (%)** | **T1 (45)**  **No. of respondents**  **Yes (%)** |
| --- | --- | --- | --- |
| Attended support group | 0 (0) | 2 (4) | 6 (13) |
| Would like to attend support group | 17 (29) | 15 (28) | 8 (18) |
| Are unaware of stroke support groups | 13 (22) | 11 (20) | 2 (4) |

**Table X.** Codes, categories and higher order categories at T0 and T2

| **Participant responses T0** | **ID** | **Codes at T0** | **Categories at T0** | **Higher Order Categories** | **Categories at T2** | **Codes at T2** | **ID** | **Participant responses T2** |
| --- | --- | --- | --- | --- | --- | --- | --- | --- |
| Not responsive - Timely access to housing grant to facilitate timely discharge | 20 | Process delays affecting discharge – e.g. disabled persons grant | Process Delays - affecting timely discharge and outcomes after stroke | **Processes for Successful Transition to Home and Life After Stroke** |  |  |  |  |
| Equipment not responsive enough | 47 | Process delays affecting discharge satisfaction |  |  |  |  |  |  |
| Length of stay and waiting for tests is too long | 48 | Process delays affecting discharge |  |  |  |  |  |  |
| Need information on the process of return to driving; | 69 | Process delays affecting social participation |  |  |  |  |  |  |
| Disabled persons housing grant is too slow, and people are struggling unnecessarily because it’s so slow. | 72 | Process delays affecting discharge – e.g. disabled persons grant, impacts on outcomes |  |  |  |  |  |  |
| Not all equipment is in place before discharge which results in unnecessary stress | 74 | Process delays affecting discharge and patient outcomes - Equipment |  |  |  |  |  |  |
| Response of community teams is too slow | 74 | Responsiveness of community as part of continuity of care – when and what services will be accessed |  |  |  |  |  |  |
| chunk of saving have been used on modification | 20 | Expenses associated with satisfactory discharge – e.g. disabled persons grant | Expenses associated with process delays |  |  |  |  |  |
| Leaves family out of pocket - equipment | 47 | Expenses associated with safe and satisfactory discharge - equipment |  |  |  |  |  |  |
| Understand aspects of stroke such as tone/spasticity and how it affects you | 27 | Accessing information on entitlements and applying for same is challenging | Comprehensive Health Information for People with Stroke and their Families | **Empowering Stroke Survivors and Families through Comprehensive Health and Social Care Information** | Accessing Financial Assistance | Support with benefits and entitlements | 29 | Support with finances |
| Mind map of roles of professionals | 27 | Need to know the team |  |  |  | Support with benefits and entitlements | 41 | Medical card based on last year’s income, so I don’t qualify - leads to anxiety around finances |
| Signposting for information to decrease risk of another stroke | 38 | Need for health information |  |  |  |  |  |  |
| Validation that the person understands the information being given | 42 | Need for health information |  |  |  |  |  |  |
| Can't take everything in in hospital and now there is no information available. | 48 | Need for health information at multiple time-points |  |  |  |  |  |  |
| Medication needs to be discussed, no idea what I'm on what for - BP meds now lower than what previously on | 51 | Need for health information around meds |  |  |  |  |  |  |
| Discussion about the stroke and why it happened - face to face with me and my family. | 51 | Health information delivery, mode of delivery, to include family |  |  |  |  |  |  |
| Information about medications …….. weren't explained to the patient or family. | 68 | Need for health information delivery, to include family |  |  |  |  |  |  |
| Need information on stroke, why I had a stroke, risk of another stroke and how to avoid | 68 | Need for health information about stroke |  |  |  |  |  |  |
| Information about stroke itself | 69 | Need for health information about stroke |  |  |  |  |  |  |
| Family involved in getting information about stroke - especially when it is clear the patient does not understand the information. | 72 | Need for health information delivery, to include family who support patient |  |  |  |  |  |  |
| The initial information piece is missing - why the stroke happened. | 72 | Need for health information about stroke |  |  |  |  |  |  |
| Partnership to decide when homecare should come, not accommodating for persons individual preferences | 47 | Needs based homecare | Partnership in goal setting and decision making | **Navigating Stroke Journeys Together** |  |  |  |  |
| Concrete plan with goal setting and targets. This needs to be a partnership, identify the problems together, and solve problems together | 57 | Goal setting in partnership |  |  |  |  |  |  |
| Goal setting needs to be realistic (positive but no fibs | 57 | Goal setting – realistic but motivating |  |  |  |  |  |  |
| Need to feel part of the transition in terms of discharge home. | 64 | Decision making in partnership |  |  |  |  |  |  |
| Sense of being a burden disabling- fostering independence is key | 34 | Need for fostering independence to decrease sense of burden | Emowering Self Management for People with Stroke |  |  |  |  |  |
| Guidance on self-management after stroke | 56 | Education on self-management |  |  |  |  |  |  |
| Need help building confidence, getting self-management support and practical tools - this would support emotionally | 57 | Education on self-management  Self-efficacy linked with emotional support |  |  |  |  |  |  |
| Lack of shared plan of action between acute and community services | 27 | Lack of communication and information sharing affecting continuity of care | Effective Communication and Information Sharing for Individualised Healthcare | **Effective Communication and Information Sharing for Individualised Healthcare** | Improving Communication and Information Sharing in Health Care for People with Stroke | Communication and information sharing across services | 12 | Consistent communication across services |
| Advice on adapting home to meet needs | 47 | Need for environmental Information - home adaptations |  |  |  | Information sharing on community based resources | 29 | Signposting for what is available in the community |
| Timely information needs to be given to the GP | 51 | Information sharing as part of continuity of care |  |  |  | Need for health information | 34 | Lack of information about stroke |
| BP meds now lower than what previously on and my GP didn't know I had a stroke". | 51 | Health information for safety  Information sharing as part of continuity of care |  |  |  | Lack of information sharing affecting continuity of care | 55 | GP awareness about the stroke |
| Dr did not know I had had a stroke when I went to see him | 55 | Information sharing as part of continuity of care |  |  |  |  |  |  |
| Communication between discharge prescription and pharmacist - there is a risk to safety with poor communication | 55 | Information sharing as part of continuity of care and safety |  |  |  |  |  |  |
| Uncertainty around when community services are going to kick in - PT/OT, knowing if they are on the system | 56 | Information sharing as part of continuity of care – when and services will be accessed |  |  |  |  |  |  |
| Prescription letter/discharge report needs to go sooner | 56 | Information sharing as part of continuity of care |  |  |  |  |  |  |
|  |  |  |  |  |  |  |  |  |
| Communication is key between acute and community services | 68 | Communication for continuity of care |  |  |  |  |  |  |
| Need information on follow up services, who they are and when they might start | 71 | Need for information sharing |  |  |  |  |  |  |
| Not enough preparation for discharge, discharge is rushed. | 73 | Discharge planning |  |  |  |  |  |  |
| Lack of consistency with homecare providers | 46 | Needs based homecare | Access to Ongoing Rehabilitation, Social Care and Social Support | **Comprehensive Whole-Systems Approach to Rehabilitation and Recovery** | Comprehensive Systems Based Approach to Rehab and Recovery | Systems approach to rehab and recovery | 1 | Community based exercises |
| Specific rehab beds for post-acute care. | 47 | Access to community rehabilitation that meets needs |  |  |  | Systems change around benefits and entitlements | 42 | Medical card based on last year’s income so I don’t qualify - leads to anxiety around finances |
| Get taxis which is an increased expense | 55 | Access to transport |  |  |  | Systems change around driving assessment | 55 | Issues around driving, highlighting the importance of the screening tool used in hospital, giving a sooner appointment for reviewing or allowing to be screened again, a plan for driving made at hospital discharge |
| Increased support at home for a while. | 56 | Need for home care |  |  |  | Info re access to ongoing rehabilitation, even private | 57 | Directory of physiotherapists with neurological background |
| infrastructure in place to help you cope while you are trying to problem solve - i.e. care support etc. | 57 | Need for social support to foster independence |  |  |  | Access to social care to cover practical activities | 71 | Home help to support food preparation |
| Early supported discharge (ESD) needs to be longer | 60 | Needs based rehabilitation for as long as required |  |  |  | Need for systems approach to rehab and recovery | 77 | Community- based exercises |
| Options for getting support at home ….. Someone to keep an eye on you. | 64 | Need for home support, monitoring |  |  | Access to rehabilitation | Access to rehabilitation | 2 | Fatigue management |
| Need for more psychological support | 73 | Emotional needs of person with stroke |  |  |  | Access to rehabilitation | 29 | More physical intervention and Access to OT |
| Medical card application – upsetting and demoralising | 27 | Accessing information on entitlements and applying for same is challenging | Tailored Financial Support |  |  | Access to rehabilitation | 55 | Access to ongoing rehabilitation |
| Social Worker or someone to help with social welfare and financial support. Needs to be available early | 58 | Accessing information on entitlements and applying for same - timely |  |  | Accessing Peer Support and Companionship | Peer support | 1 | Information on people with similar issues to mine |
| Someone to work through entitlements with you from an early stage | 60 | Accessing information on entitlements and applying for same - timely |  |  |  | Peer support | 29 | Access to peer support |
| Information about financial supports to know entitlements early. | 69 | Accessing information on entitlements and applying for same - timely |  |  |  | Companionship | 42 | Companion who is age appropriate and individualised to patient needs and interests |
| Map of locally accessible places | 27 | Need for Information to support social participation | Peer Support, Voluntary Organisations and Community Groups for Individual Assistance and Information Exchange |  |  | Peer support | 77 | Information on people with similar issues to mine |
| Peer groups within the acute system for information and support | 41 | Need for peer support |  |  | Holistic Approach To Recovery | Holistic approach to recovery | 12 | Should consider the persons medical needs as a whole |
| Information on the Irish Heart Foundation* - heard rumours of what’s available but nothing concrete | 48 | Whole systems approach to information to support – voluntary stroke organisations |  |  |  |  |  |  |
| Someone to talk to, peer company | 50 | Social prescribing |  |  |  |  |  |  |
| Need information on driving and the process of return to driving; | 69 | Information and support for social participation |  |  |  |  |  |  |
| Need information about support groups | 69 | whole systems approach to information to support – voluntary organisations |  |  |  |  |  |  |
| Need for more clarity around driving and a more practical way to assess driving early on | 74 | Information and support for social participation |  |  |  |  |  |  |
| Holistic Care | 56 | Holistic Care | Holistic Approach To Recovery |  |  |  |  |  |
| Families to be part of the recovery | 40 | Need to Engage family in recovery plans | Family Engagement | **Empowering Families** | Education for Carers and Building Confidence in Stroke Survivors’ Autonomy | Family education on capacity of stroke survivor | 42 | Education for families in terms of building their confidence around stroke survivors abilities to make appropriate decisions for themselves |
| Caregivers need access to counselling | 41 | Emotional needs of caregivers |  |  |  | Education to carers to enable them to meet needs of individuals | 75 | Need to give carer guidance on their role |
| Knock on effect when the person who had the stroke was a carer already. Now they are reliant on other people for transport for themselves and their partner, they are a burden | 55 | Impact on an already challenged family system  Sense of burden |  |  |  |  |  |  |
| Information …… weren't explained to the patient or family. | 68 | Need to engage family in recovery plans |  |  |  |  |  |  |
| Family is not engaged/brought in enough in the acute process | 72 | Need to engage family in recovery plans |  |  |  |  |  |  |
| Family involved in getting information about stroke - especially when it is clear the patient does not understand the information. | 72 | Need for health information delivery, to include family who support patient |  |  |  |  |  |  |
| Family of person with stroke need respite and counselling when the patient gets home. | 72 | Emotional needs of caregivers |  |  |  |  |  |  |
| Family of person with stroke need respite and counselling when the patient gets home. | 74 | Emotional needs of caregivers |  |  |  |  |  |  |
| Key workers across acute and community - information point, be able to signpost and follow-up with services | 41 | Key works as part of continuity of care – role in information, signposting and communication btw services | Keyworker Role as Bridge between Healthcare Settings and Person with Stroke / Family | **Keyworker / Case manager Role: A Bridge between Healthcare Settings and Person with Stroke / Family** | Case Co-ordinator to Facilitate Transitions | Case manager to coordinate between services | 25 | Case manager to act as coordinator between services |
| Keyworker required to follow up and check in | 42 | Key workers as part of continuity of care |  |  |  | access to “someone” to ask questions about residual issues for at least 6 months after stroke | 24 | Access to “someone” to ask questions about residual issues for at least 6 months after stroke |
| Link person between the acute and community to field questions | 48 | Key worker as part of continuity of care |  |  |  | access someone to ask questions about residual issues for at least 6 months after stroke – a link person | 42 | Access someone to ask questions about residual issues for at least 6 months after stroke – a link person |
| Someone to ask questions of at home - a link between hospital and community | 60 | Key worker as part of continuity of care |  |  |  | Someone to introduce to CROI etc. and facilitate seamless transition | 76 | Someone to introduce to CROI* etc. and facilitate seamless transition |
| Link person between the hospital, community, patient and family | 68 | Key worker as part of continuity of care |  |  |  |  |  |  |
| Face to face reviews | 39 | Review face to face | Timely Monitoring and Review of Residual Needs | **Comprehensive Monitoring and Support for Residual Needs and Long-term Stroke Recovery** | Monitoring and Support for Long-term Recovery | Monitoring | 2 | Role of the GP in monitoring |
| 3-months to first review is too long | 42 | Need for more timely review – medical or other |  |  |  | Ongoing monitoring | 12 | Follow up needs to be longer than 3 months |
| Need to be called back sooner to OPD | 56 | Need for more timely review – medical or other |  |  |  | Access to “knowledgeable individual”” as part of continuity of care | 24 | Access to “someone” to ask questions about residual issues for at least 6 months after stroke |
| Timely review after hospital discharge to identify residual needs | 65 | Need for more timely review |  |  |  | Access to “knowledgeable individual”” as part of continuity of care | 42 | Access someone to ask questions about residual issues for at least 6-months after stroke – a link person |
| Too long to wait for 3-4 month review | 74 | Need for more timely review – medical or other |  |  |  | Need for more timely, regular and holistic review | 41 | More regular review with GP or stroke team, monthly for 6 months |
|  |  |  |  |  |  | Need for needs based review by appropriate team, "to do a final signoff"- check in by ESD  Role in grieving process - acceptance | 58 | Follow-up after 3-months by ESD team, for review and residual needs assessment and signposting as appropriate - "to do a final signoff", to be able to ask questions about symptoms that emerge in LT, to help adjust and accept any mild deficits. This would help you to accept the stroke, role in grieving process |
|  |  |  |  |  |  |  | 76 | Check in service or review service with MDT |

*The Irish Heart Foundation and CROI are registered charities, who play a significant role in supporting individuals and families in Ireland who have been affected by stroke and heart disease.

**Processes for Successful Transition to Home and Life After Stroke** highlights the negative impact of process delays on various aspects of transition to home and life after stroke. The slow access to housing grants, and delays in obtaining necessary equipment before discharge, leads to prolonged length of hospital stay, financial strain, and unnecessary stress on the individual and their family. Moreover, waiting times for tests e.g. MRI, added to the overall problem, hindering timely and efficient discharge. This theme emphasises the financial stress on people with stroke when the current disability benefit system allocates support based on income rather than individual circumstances and requirements. This results in further anxiety for the person with stroke and their family.

**Empowering Stroke Survivors and Families through Comprehensive Health and Social Care Information** highlights a need for comprehensive health and social care information and support to people with stroke and their families at transition to home, to facilitate understanding of stroke and it’s impairments, medication usage, and how to prevent future strokes; to promote social participation including facilitating a smooth return to driving; and to enable early access to financial assistance, by providing guidance on entitlements, and support in applying for and accessing benefits.

**Navigating Stroke Journeys Together** highlights a need for a collaborative approach between individuals and healthcare professionals in realistic goal setting, which would promote positivity, and decision-making for transition to home. The theme addresses participants need for self-management strategies so people with stroke can take control of their care, enhance their abilities, and reduce sense of burden. Participants felt that bridging these gaps would impact on recovery and emotional well-being.

**Effective Communication and Information Sharing for Individualised Healthcare** highlights the gaps in communication and information sharing within the healthcare system, both between health services and patients, and across different healthcare providers. Key challenges include sharing timely information about stroke, medication, and ongoing needs, which impacts patient safety and continuity of care. At 6-months the theme extends to being responsive to individuals’ residual needs and sharing information about community resources.

**Comprehensive Whole-Systems Approach to Rehabilitation and Recovery** highlights a need for clear follow-up pathways including tailored and accessible rehabilitation, increased social care services, and referral to voluntary groups and community organisations to help individuals recover and build capacity. It documents inconsistencies in homecare provision and a gap in dedicated rehabilitation beds and access to ongoing community rehabilitation. Respondents call for extended ESD programmes, access to specialised therapy including psychological support, fatigue management and exercise prescription. At all time-points respondents require tailored support to address financial anxiety, driving concerns, and access to home help. The need for signposting to voluntary groups and community organisations who can bridge gaps, offer peer support, and foster community and understanding is further discussed at 6-months.

**Empowering Families:** highlights the need to engage and support the families of people with stroke, including them in their loved one’s recovery, and acknowledging the burden and overwhelm they face. It highlights their need for emotional support, and access to respite and counselling. At 6-months the theme evolves to a need to tailor education to increase understanding among caregivers of the needs and capacity of people with stroke.

**Keyworker / Dedicated Case Manager Role: a Bridge between Healthcare Settings and Person with Stroke / Families** highlights a desire for a liaison role to provide support, information, and continuity of care throughout the healthcare journey by identifying needs, addressing gaps, providing information, and referring people with stroke to appropriate services. This theme is again reflected as 6-months with suggestions a Keyworker / Dedicated Case Manager would facilitate a seamless transitions between care stages, by coordinating healthcare services and facilitating access to support groups.

**Comprehensive Monitoring and Support for Residual Needs** **and** **Long-Term Stroke Recovery** highlights the need for timely monitoring and review to address residual needs, with the importance of face-to-face reviews specified. At 6-months, more detail emerges to emphasise timely and ongoing reviews with stroke teams, multidisciplinary teams and the General Practitioner (GP), who all have an important role in identifying residual needs, providing ongoing support, and helping individuals to accept a new life after stroke. A holistic approach to follow-ups for coordinated and effective stroke recovery is highlighted.
